# Evaluating traces of Hebbian plasticity in the *Drosophila* antennal lobe

**DOI:** 10.1073/pnas.2315790120

**Published:** 2023-12-04

**Authors:** André Ferreira Castro, Scott Wilson, Albert Cardona

**Affiliations:** ^a^Neurobiology Division, Medical Research Council Laboratory of Molecular Biology, Cambridge CB2 0QH, United Kingdom; ^b^Department of Physiology, Development and Neuroscience, University of Cambridge, Cambridge CB2 3DY, United Kingdom

The assembly of functional neuronal circuitry relies on the precise temporal modulation of synaptic weights. An elegant computational analysis by Chapochnikov et al. (1) elucidated a robust circuit motif in the *Drosophila* melanogaster larval antennal lobe (AL) that can extract input features, rendering stimulus representations more efficient. The authors proposed that such synaptic organization could potentially emerge autonomously through Hebbian plasticity in the AL. However, the degree of activity-dependent Hebbian plasticity in the larval AL remains to be clarified.

**Fig. 1.**
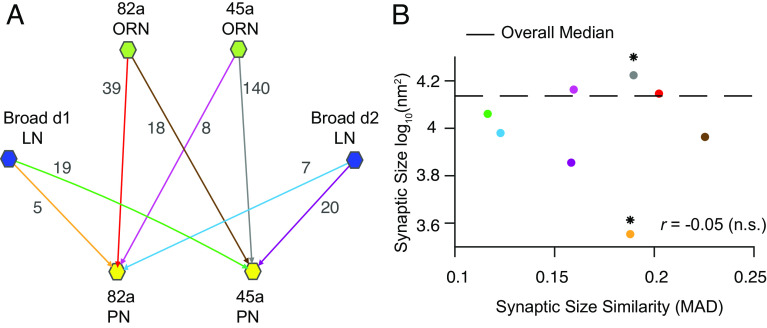
Circuit connectivity and connectomic mapping of the plasticity-consistent circuit fraction. (*A*) Scheme of the subcircuit of AL studied, with available ORN (green hexagon)-PN (yellow hexagon) and LN (dark blue hexagon)-PN connections. Lines and arrowheads represent connections found in AL, where each line and arrowhead have a unique color. The number of synaptic areas available per connection is indicated next to the corresponding line and arrowhead. (*B*) Distribution of median synaptic size (log-transformed data) and synaptic size similarity, with synaptic size similarity quantified by the median absolute deviation (MAD) for all synaptic pairs; each dot corresponds to connection pair. Color coding as in *A*. The star sign indicates statistical significance on synaptic size differences, *P* < 0.0125, after Bonferroni’s correction. Pair-wise statistical analysis using permutation and Kolmogorov–Smirnov tests.

To investigate this, we leveraged a connectomics dataset to search for traces of plasticity in the larval AL (2). This structural dataset consisted of 256 synapses, across four olfactory receptor neurons (ORN) to projection neurons (PN) excitatory connections, and four local neurons (LN) to PN inhibitory connections (Fig. 1*A*). While no available dataset perfectly matches the ORN-LN and LN-LN connections from ref. (1), analyzing ORN-PN data may reveal Hebbian plasticity traces in the AL. Thus, we aimed to map the relationship between synaptic size, a known anatomical correlate of synaptic strength (3), and synaptic size similarity across these connections. We hypothesised that in case Hebbian plasticity was at play in modulating synaptic size, long-term potentiation (LTP) would cause synapses from the same connection to become stronger and relatively more similar in weight due to weight saturation, and long-term depression (LTD) would lead joint synapses to become weaker and relatively more dissimilar in weight (4).

To test these predictions, we compared the median synaptic sizes and synaptic size similarities, by computing the median absolute deviation (MAD), across all connections in the dataset (Fig. 1*B*). Our findings revealed a nonsignificant (n.s.) inverse relationship between these variables, with a correlation *r* = −0.05 (permutation tests, *P* = 0.9). When analyzing individual synapses, no connection subtype demonstrated significant oversimilarity (permutation tests, *P* > 0.0125, after Bonferroni’s correction). Surprisingly, this was even the case in connections that showed a significantly larger (45a ORN-PN median = 4.22 log_10_nm^2^, *P* < 0.001; MAD = 0.189, *P* = 0.12) or smaller median synapse size (Broad d1- 82a PN median = 3.55 log_10_nm^2^, *P* < 0.01; MAD = 0.187, *P* = 0.81; Fig. 1*B*). Contrary to expectations, our results indicate that synapses in this subcircuit of the AL do not display traces of Hebbian plasticity consistent with LTD or LTP.

In summary, our analysis of synaptic characteristics within a small fraction of the AL circuit did not align with traces of Hebbian or activity-dependent synaptic plasticity. Nonetheless, we encourage a more comprehensive analysis of AL connection types to validate our findings.
